# Elevated Levels of Eotaxin-2 in Serum of Fibromyalgia Patients

**DOI:** 10.1155/2018/7257681

**Published:** 2018-05-13

**Authors:** Victoria Furer, Eyal Hazan, Adi Mor, Michal Segal, Avi Katav, Valerie Aloush, Ori Elkayam, Jacob George, Jacob N. Ablin

**Affiliations:** ^1^Tel Aviv Sourasky Medical Center and the Sackler Faculty of Medicine, Tel Aviv University, Tel Aviv, Israel; ^2^Tel Aviv Sourasky Medical Center, Tel Aviv, Israel; ^3^ChemomAb Ltd., Tel Aviv, Israel; ^4^Kaplan Medical Center, Rehovot, Israel

## Abstract

FMS patients demonstrate an altered profile of chemokines relative to healthy controls (HC). Eotaxin-2 is a potent chemoattractant distributed in a variety of tissues. The aim of the study was to compare serum levels of eotaxin-2 between FMS patients and HC and to examine a potential correlation between eotaxin-2 levels and clinical parameters of FMS. *Methods*. 50 patients with FMS and 15 HC were recruited. Data on the severity of FMS symptoms and depression were collected. Serum levels of eotaxin-2 (ELISA) were determined in all participants. High-sensitive CRP (hs-CRP) was measured in the FMS group. *Results*. The FMS cohort included predominantly females (84%), mean age of 49, and mean disease duration of 6 years. FMS patients exhibited significantly higher eotaxin-2 levels (pg/ml) versus HC: 833 (±384) versus 622 (±149), *p*=0.04. Mean hs-CRP level among FMS patients was 4.8 ± 6 mg/l, a value not indicative of acute inflammation. No correlation was found between eotaxin-2 and hs-CRP levels. No correlation was found between eotaxin-2 and severity measures of FMS or depression. *Conclusion*. Eotaxin-2 does not appear to be a candidate for a disease activity biomarker in FMS. Further research is warranted into the role of this chemokine in the pathophysiology of the FMS.

## 1. Introduction

Fibromyalgia syndrome (FMS) is a highly prevalent chronic pain syndrome characterized by widespread pain and other somatic symptoms, including fatigue, sleep disturbances, cognitive dysfunction, and depression [1]. The diagnosis of FMS is based on clinical grounds [2], and despite many attempts to identify objective biomarkers for FMS, no such well-validated biochemical marker for either diagnosis or severity has emerged. While the pathogenesis of FMS remains incompletely understood, one leading paradigm is that of pain centralization, an increase of the processing of pain within the central nervous system (CNS) [3].

Recent studies suggest that cytokines may play a role in the pathogenesis of FMS, in particular, chemotactic cytokines referred as chemokines [4]. Chemokines are a family of small (8–10  kDa) proteins that induce chemotaxis of inflammatory cells. Emerging evidence reveals that chemokines play a role in the physiology of the nervous system, including neuronal migration, cell proliferation, and synaptic activity [5]. Chemokines and their receptors are among the key players responsible for communication between neurons and inflammatory cells, and this crosstalk is crucial for normal neurological functioning [5]. Furthermore, chemokines seem to contribute to a reciprocal interaction between neurons, glia, and microglia in a so-called “gliopathy,” that is, activation of glial cells and neuroglial interactions as a basis for chronic pain [6–8]. Chemokines participate in synaptic transmission and in the formation of second-messenger systems in neurons and glial cells, favoring the noxious process. Chemokines enhance sensitivity to pain by direct action on chemokine receptors expressed in the entire pain pathway, from peripheral nerves to the dorsal ganglia and spinal cord. Simultaneously, they regulate the inflammatory response by acting on elements of the nervous system [5].

Since pain is the salient symptom of FMS, one may consider that, as modulators of nociception, certain chemokines may be involved in the pathophysiology of this syndrome. Increased levels of inflammatory cytokines and chemokines in serum [4, 9, 10] and cerebral spinal fluid [11] have been recently reported in cross-sectional studies in FMS cohorts. Subsequently, a prospective study by Wang et al. has not only confirmed the finding of increased circulating levels of cytokines in FMS, but also suggested a potential cytokine response to a therapeutic intervention [12, 13]. After 6 months of multidisciplinary pain therapy, baseline serum level of interleukin-8 (IL-8) reduced nearly to the normal range in correlation with a reduction in pain intensity. Ang et al. have further demonstrated that the level of pain in FMS corresponds to circulating chemokines levels, with uptrending levels of monocyte chemotactic protein-1 (MCP-1) and IL-8 in parallel with increasing pain severity over time [14].

Nonetheless, the relationship between the chemokine-cytokine network and FMS has yet to be clarified. Eotaxin-2 (CCL24), a member of the CC chemokine family, is a potent chemoattractant for eosinophils, basophils, and lymphocytes, distributed in a variety of tissues, including human brain [15]. To our best knowledge, no studies previously examined the eotaxin-2 profile in FMS patients. Thus, we conducted a case-control study to determine levels of circulating eotaxin-2 and high-sensitive C-reactive protein (hs-CRP) in FMS patients compared to healthy controls (HC). We further examined the relationship between these potential biomarkers and FMS severity.

## 2. Methods

50 patients suffering from primary FMS were consecutively recruited through the Rheumatology Clinic at the Tel Aviv Sourasky Medical Center. 15 healthy subjects were recruited as healthy controls (HC). Upon recruitment, patients were examined by a physician to verify the diagnosis of FMS and screened for alternative diagnoses such as inflammatory joint disease. Patients with a known diagnosis of inflammatory joint disease/chronic kidney disease/chronic liver disease/heart failure/diabetes mellitus/active malignancy were excluded from the study. Patients currently treated with immune-suppressive medications, including steroids, were excluded from the study. The diagnosis of FMS was verified according to the American College of Rheumatology (ACR) updated diagnostic criteria [2]. Patients subsequently filled out questionnaires to asses and document the severity of FMS symptoms. Questionnaires included basic demographic data (age, sex, smoking status, weight, height, use of medications, comorbidities, and previous medical history), widespread pain index (WPI), documenting extent of widespread pain, symptoms severity score (SSS), documenting severity of associated symptoms, Fibromyalgia Impact Questionnaire (FIQ), and Beck Depression Inventory (BDI) for evaluation of depression. Blood specimens for Eotaxin-2 and hs-CRP were drawn, separated, aliquoted, and stored frozen at −20°C until analysis. Eotaxin-2 was measured using ELISA. The study was approved by the Institutional Review Board of the Hospital, and all patients provided written informed consent.

## 3. Statistical Analysis and Data Processing

Data were statistically analyzed with SPSS (version 20; IBM, Armonk, New York, NY, USA). Before analysis, residuals were tested for normal distribution (Shapiro–Wilk test) and equality of variance (Levene's test). Nonparametric tests were used where appropriate. Group comparisons were calculated using Student's independent *t*-test (parametric), Kruskal–Wallis test (nonparametric) or Pearson's chi test for categorical variables. According to the underlying hypotheses, a two-tailed test was performed. The significance level was set to *p*=0.05. Values are given as means ± standard deviations (SD).

## 4. Results

Fifty-four patients suffering from FMS and 15 HC were enrolled in the study. Four patients were excluded due to impaired glucose tolerance treated pharmacologically (*n*=2) or due to missing data (*n*=2). The FMS cohort included predominantly females (84%) of 49 ± 14.6 years of age, body mass index (BMI) of 26.8 ± 5.1, and disease duration of 6 ± 5.5 years. Thirty percent of FMS patients were smokers. Half of the patients were unemployed. Thirty-two percent of patients stated incapability to work due to FMS. HC cohort included subjects of 37.5 ± 12.6 years of age, with a similar gender representation (53% females and 47% males) and BMI 22.6 (±3.3).

**Figure 1:**
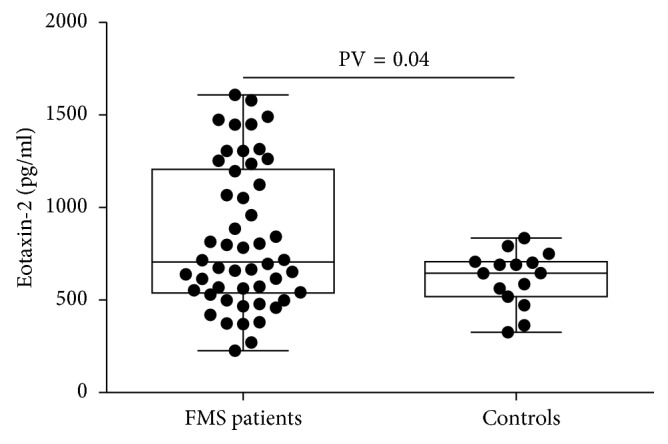
Eotaxin-2 levels in serum of patients with FMS and healthy controls.

The levels of the mediators along with indices of disease activity in FMS are summarized in Table 1. FMS patients exhibited significantly higher eotaxin-2 levels as compared to healthy controls, 833 (±384) versus 622 (±149), *p*=0.04, respectively, as presented in Figure 1. No significant gender-based difference was found in the mediators' levels or disease severity indices.

When examining the correlation between FMS severity indices and levels of eotaxin-2, no significant correlation was found. There was also no significant correlation between the parameters of FMS severity and hs-CRP. The lack of correlation between eotaxin-2 and hs-CRP is noteworthy. The majority of FMS patients had normal range of hs-CRP levels; however, the average level of hs-CRP was slightly elevated in FMS patients (4.81 mg/l). As expected, a positive correlation was found between hs-CRP and BMI (*r*=0.44,  *p*=0.02), but not for eotaxin-2 levels and BMI (*r*=0.06,  *p*=0.8). Smoking was not found to correlate neither with eotaxin-2/hs-CRP levels nor with disease severity indices.

## 5. Discussion

The present pilot study is the first one to report significantly increased circulating levels of eotaxin-2 in serum of FMS patients, compared with healthy controls, with no direct association between eotaxin-2 levels and FMS severity indices. This finding is consistent with the accumulating evidence regarding a distinct cytokine profile in FMS patients, further supporting the hypothesis of cytokines playing an important role in FMS pathophysiology [4, 10, 12–14].

Eotaxins are C-C motif chemokines first identified as potent eosinophil chemoattractants. They facilitate eosinophil recruitment to sites of inflammation in response to parasitic infections, as well as in allergic and autoimmune diseases such as asthma, atopic dermatitis, and inflammatory bowel disease [16]. The eotaxin family currently includes three members: eotaxin-1 (CCL11), eotaxin-2 (CCL24), and eotaxin-3 (CCL26). Despite having only ∼30% sequence homology to one another, each was identified based on its ability to bind the chemokine receptor, CCR3. Increased serum eotaxin-1 levels have been reported in FMS patients [4, 10] and in patients with neurodegenerative diseases, including Alzheimer's disease, amyotrophic lateral sclerosis, Huntington's disease, and secondary progressive multiple sclerosis [17]. Since eotaxin-1 is capable of crossing the blood-brain barrier of normal mice, it is plausible that eotaxins generated in the periphery may exert physiological and pathological actions in the CNS.

Eotaxin-2 is a potent chemoattractant that binds to CCR3 for intracellular messaging. A variety of cells including respiratory epithelial cells, bronchial smooth muscle cells, vascular endothelial cells, fibroblasts, monocytes, helper T cells, and basophils express CCR3 and respond to eotaxin-2 stimulation [18], implicating a role in cellular communication. Interestingly, inhibition of eotaxin-2 by antibodies conveys an efficient protective effect in experimental arthritis [19] although the pathogenic mechanism is still unclear. While no data on the levels of eotaxin-2 in the FMS are available in the literature, patients with the related chronic fatigue syndrome demonstrate a distinct cytokine/chemokine plasma profile, including elevated levels of eotaxin-2 compared to healthy controls [20].

In order to address the inflammatory status of the FMS cohort and to rule out the possibility that eotaxin-2 levels might represent a nonspecific acute phase reactant, hs-CRP levels were tested. The majority of the FMS cohort had indeed normal levels of hs-CRP. Yet, the mean level of hs-CRP was slightly elevated among the FMS patients, a value considered to represent an increased cardiovascular risk but not a state of acute inflammation. hs-CRP levels did not correlate with eotaxin-2 levels. Consistently, no correlation between hs-CRP and eotaxin-2 levels was found in a healthy and overweight Japanese cohort [21].

In the present study, there was no correlation between hs-CRP and FMS severity measures. Interestingly, a number of studies reported elevated CRP levels in FMS patients [9, 12, 22] though these studies did not examine the hs-CRP levels. In our study, there was a moderately positive correlation between hs-CRP and BMI. In the general population, a positive correlation between elevated BMI, visceral and abdominal subcutaneous adipose tissue, and waist circumference in respect to hs-CRP levels has previously been reported [23]. Thus, increased BMI among the FMS cohort in our study may explain the slightly elevated levels of mean hs-CRP.

No association between eotaxin-2 levels and measures of disease severity were found in the present study, thus not supporting a role for eotaxin-2 as a biomarker of disease severity.

A number of limitations of the current study should be pointed out. While our study focused on eotaxin-2, this chemokine acts in close interaction with a large number of additional mediators, which has not been investigated in the present cohort. Further, hs-CRP levels were measured only in the FMS and not healthy cohort. In view of our findings, it would be intriguing to further scrutinize additional chemokines and to sketch a more extensive map of chemokine aberrations in FMS. Identifying such individual patterns of chemokines expression might eventually lead to more precise characterization of individual patients, ultimately aiding in therapeutically targeting specific chemokines, as a strategy for individualized treatment. Another limitation of the study lies in the relatively limited sample size of patients and controls. Going back to the proposed hypothesis regarding the role of chemokines in a chronic pain related gliopathy, future research may be directed at cross-correlating alterations in chemokine levels (such as found in the current study) with advanced functional imaging techniques which may become available in order to study such a gliopathy [24].

In conclusion, in the current study, serum eotaxin-2 levels were investigated for the first time in FMS patients and found to be significantly increased compared to healthy controls. While eotaxin-2 levels did not correlate with FMS disease severity, this finding calls to search new pathogenetic mechanisms of the syndrome. A study of eotaxin-2 levels in the cerebrospinal fluid in relation to the level of sleep interruption and fatigue intensity may be of interest. Further studies of additional cytokines and especially chemokines are indicated in order to pinpoint a biochemical marker for FMS disease severity, while additional research is also necessary in order to uncover a possible pathogenetic role of eotaxin-2 in FMS and in order to evaluate its potential role as a therapeutic target.

## Figures and Tables

**Table 1:** Levels of the mediators and indices of disease activity in FMS cohort (*N*=50).

	Mean	Std. deviation
Eotaxin-2 (pg/ml)	833.82	384.67
hs-CRP (mg/l)	4.81	6.02
WPI	12.5	4.21
SSS	9.08	1.98
FIQ	63.88	16.81
BDI	20.00	11.05

hs-CRP, high sensitivity C-reactive protein; WPI, widespread pain index; SSS, symptoms severity score; FIQ, fibromyalgia impact questionnaire; BDI, beck depression inventory.
